# Case Report: Sufficient pelvic floor muscle function can retain acceptable postoperative defecation function after *in-situ* anal reconstruction surgery in patients with ultra-low rectal/anal cancer

**DOI:** 10.3389/fsurg.2026.1762110

**Published:** 2026-03-03

**Authors:** Li Lin, Yihui Lei, Guoyan Liu

**Affiliations:** 1 Department of Gastrointestinal Surgery, Xiang'an Hospital of Xiamen University, School of Medicine, Xiamen University, Xiamen, Fujian, China; 2 Department of Gastrointestinal Surgery, Xiamen Humanity Hospital, Xiamen, Fujian, China; 3 The School of Clinical Medical, Fujian Medical University, Fuzhou, Fujian, China; 4 School of Pharmaceutical Sciences, Xiamen University, Xiamen, Fujian, China

**Keywords:** acceptable postoperative defecation function, anal canal cancer, case report/case series, *in-situ* anal reconstruction, ultra-low rectal cancer

## Abstract

**Background:**

Patients with ultra-low rectal cancer/anal canal cancer generally undergo abdominoperineal resection with sigmoid colostomy. Patients commonly experience poor quality of life postoperatively, often feeling that their dignity is compromised. Some patients are even willing to forego treatment rather than lose their anus. Surgical approaches to fulfill the treatment aspirations of these patients require further investigation.

**Case presentation:**

Three patients with rectal/anal cancer who underwent combined procedures of partial pelvic floor muscle resection and *in situ* anal reconstruction were included. All patients underwent combined partial pelvic floor muscle resection and *in situ* anal reconstruction by the same surgeon. At 24 months postoperatively, the Wexner score and Low Anterior Resection Syndrome score of patient 1 were 7 and 18, and she reported effective control of bowel movements. The Wexner score and Low Anterior Resection Syndrome score of patient 2 were 12 and 37. His bowel function had significantly improved, with only one to two incidents of fecal incontinence per week, which did not substantially impact his daily life. The Wexner score and Low Anterior Resection Syndrome score of patient 3 were 16 and 37. He could perceive the urge to defecate and suppress it for up to 20 s.

**Conclusion:**

Patients with good pelvic floor function who strongly refuse permanent stoma can undergo a combination of partial pelvic floor muscle resection and *in situ* anal reconstruction.

## 1 Introduction

Colorectal cancer is the third most prevalent malignancy worldwide, accounting for approximately 10.0% of total cancer cases (1). Its incidence is progressively leaning toward younger demographics, evident in the proportion of cases among individuals below 55 years of age in the United States, which increased from 11% in 1995 to 20% in 2019 (2). Young patients often exhibit a profound demand for postoperative dignity and defecation. Since the introduction of intersphincteric resection (ISR) by Heseker et al. in 1994 (3), ISR has emerged as the ultimate anal-preservation surgery for distal rectal cancer. Its surgical indications have expanded to include cases in which the lower edge of the tumor is <4 cm from the anal verge (4). However, up to the present time, involvement of the external anal sphincter (EAS) or levator ani (LA) remains a contraindication for ISR surgery (4). Additionally, owing to the frequent association of ultra-low rectal cancer (ULRC) with anal canal involvement, following the treatment guidelines recommended by the National Comprehensive Cancer Network entails performing abdominoperineal resection (APR) procedure (5). This results in an inability to preserve the anus, which is unacceptable for certain patients. They believed that losing the anus would compromise their dignity, and that having a stoma bag attached to the abdominal wall would disrupt their daily lives and exacerbate psychological distress. Their concerns included the potential for fecal odor leaking from the stoma bag and affecting others, the possibility of bag rupture if not changed promptly, and the fear of social isolation as others may distance themselves because of the presence of the stoma bag. Smith et al. (6) have also indicated that some patients undergoing stoma surgery suffer from significant physical and psychological distresses. Therefore, they would forgo treatment altogether rather than relinquish their anus. Thus, this study aimed to investigate whether *in situ* anal reconstruction could preserve the anus in patients with ULRC/anal canal cancer (ACC) while ensuring radical resection of the cancer.

Current research indicates that the functionality of pelvic floor muscles (PFMs) decisively controls the quality of defecation function (7–9). PFMs are mainly composed of the internal anal sphincter (IAS), EAS, and other together. Among these, the IAS is an involuntary muscle that adjusts closure and relaxation in response to rectal content, whereas the EAS and LA are voluntary muscles that can contract through self-aware control, thus resisting pressure within the rectum during defecation (10). The puborectalis muscle (PM) serves as the sensory center for the urge to defecate (11, 12). The two legs of the PM converge at the posterior aspect of the anorectal junction, forming a sling that exerts forward traction at the anorectal junction, thus creating an anorectal angle (10). The PM controls the anorectal angle. During contraction, the PM compresses the rectum from the side, causing it to move forward and upward, thereby narrowing the anorectal angle. This mechanism resembles that of a gate, which prevents fecal matter from descending into the anal canal (10). Morgan et al. (13) concluded that as long as the morphology of the PM is intact, some degree of defecation can still be preserved. The PM plays a pivotal role in defecation.

The present study confirms that the functional strength of the PM is directly proportional to its thickness (14). Hence, we assessed the functional strength of the PM by considering its thickness and, in conjunction with digital rectal examination (DRE), conducted a preliminary evaluation of PFM functionality. We propose that patients with ULRC/ACC with good PM function can maintain partial defecation after undergoing combined procedures of partial PFM resection with *in situ* anal reconstruction (PPFMR with ISAR). Here, we present three case reports.

## 2 Surgical approach

The patient was placed in the lithotomy position under general anesthesia, and a pneumoperitoneum was established at 12–15 mmHg. Employing the conventional 5-port technique, a 10-mm incision was made 0.5 cm above the umbilicus to create the observation port for pneumoperitoneum. A 12-mm trocar was inserted within the right anterior superior iliac spine, and three 5-mm trocars were placed at the midpoint of the line connecting the right clavicle to the umbilicus, the umbilicus to the midpoint of the line connecting the left anterior superior iliac spine, and the symphysis pubis joint.

### 2.1 Laparoscopic group

An ultrasonic knife was used to incise the sigmoid mesentery at the level of the corneal sacralia, entering and expanding the surgical plane between the Toldt's fusion fascia and Gerota's fascia. At the root of the inferior mesenteric artery (IMA) or the preserved left colic artery, the IMA and the inferior mesenteric vein were ligated. Subsequently, the dissection was continued upward to complete the mobilization of the left hemicolon. Sharp dissection was performed along the posterior rectal space, separating the mesorectum from the tip of the coccyx. The Denonvilliers fascia was opened to expose the seminal vesicles and the upper margin of the prostate/(or) posterior vaginal wall, accessing the pre-rectal space. A tunnel-like dissection was performed along the presacral space to complete total mesorectal excision, descending to expose the LA plane, while ensuring protection of the pelvic autonomic nerves. The upper margin of the tumor was confirmed, and the sigmoid colon and mesentery were severed approximately 15 cm from the proximal end of the tumor.

### 2.2 Anal group

**Figure 1:**
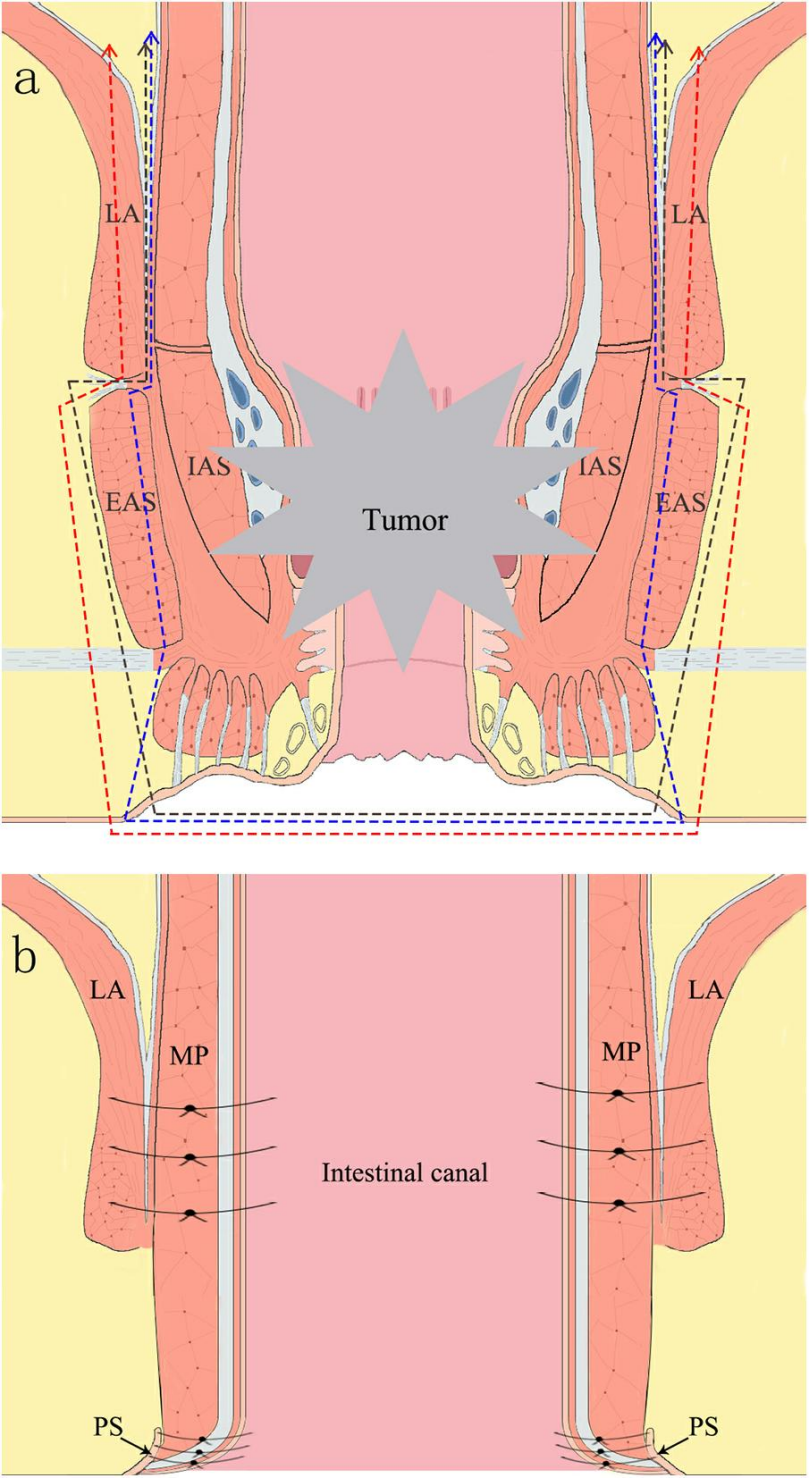
**(a)** illustration of resection scope in procedure of *in-situ* reconstruction of the anus. LA, levator ani; EAS, external sphincter muscle; IAS, internal sphincter muscle. A. The blue line represents the excision of the entire IAS and a portion of the EAS. B. The black line signifies the removal of the entire IAS and the EAS. C. The red line indicates the excision of the entire IAS and the EAS, and a segment of the puborectalis muscle. **(b)** Illustration of post-resection situation in procedure of *in-situ* reconstruction of the anus. PS, perianal skin; MP, muscularis propria; LA, levator ani muscle.

The perineal area was routinely disinfected. An incision was made along the anal margin and careful dissection was performed along the outer edge of the EAS. Gradually progressing upward within the fatty tissue situated 1 cm away from the tumor's outer margin, the tissue layers were separated until they reached the lower border of the LA. Concurrently, the laparoscopic team performed a precise sharp dissection along the plane between the PM and EAS, progressing downward. The portion of the LA invaded by the tumor was excised in harmony with the laparoscopic group to detach the rectum and anus. A schematic of the excision range is shown in Figure 1a. The excised specimen was removed from the anus and sent for pathological examination.

### 2.3 *In-situ* anal reconstruction

**Figure 2:**
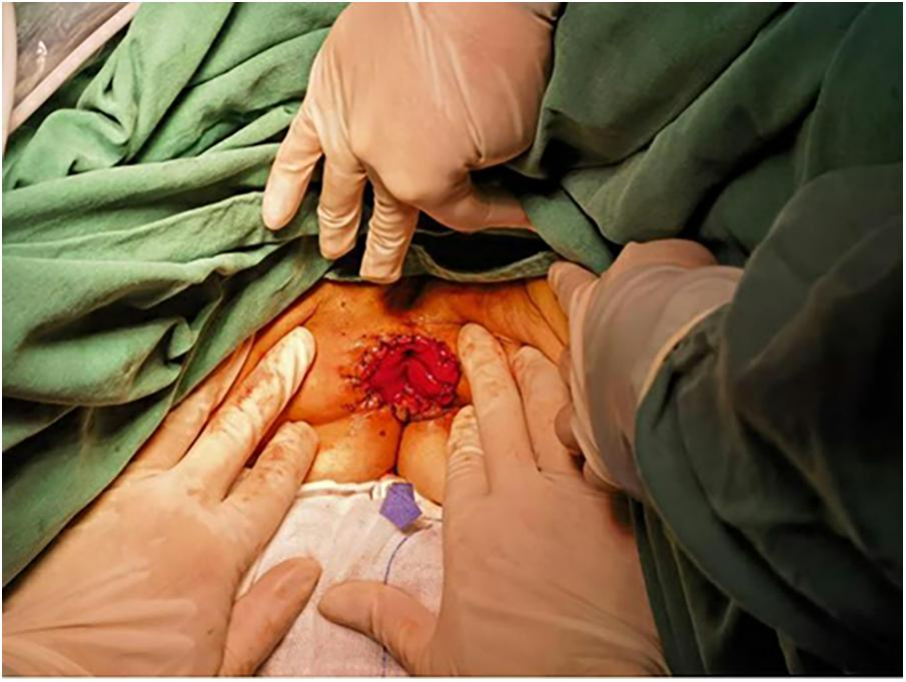
Postoperative appearance of the anal region.

The sigmoid colon was drawn out through the anus to a length of approximately 8 cm, and at a distance of 6 cm from the sigmoid colon stump, the colonic muscular layer was sutured to the lower edge of the LA at the 3, 6, 9, and 12 o'clock positions using intermittent stitches to prevent intestinal contraction or prolapse (Figure 1b). Colonic anal skin anastomosis was performed using 8–12 stitches of 3–0 absorbable sutures (Figure 2). Under laparoscopy, the intestinal tract was confirmed to be tension-free, and a drainage tube was placed.

## 3 Introduction of three cases

The inclusion criteria were as follows: PM thickness ≥ 6·5 mm for the left side and ≥4·9 mm for the right side and Oxford Modified Grading Scale ≥ grade 5; the patient strongly insisted on preserving the anus and refused stoma surgery, stating that they would rather forgo treatment altogether if preserving the anus was not viable; no pre-operative radiotherapy or chemotherapy was administered; and the ULRC/ACC invaded >1 cm below the dentate line with no distant metastasis. The baseline data of the three selected patients are shown in Table 1.

**Table 1:** Basic characteristics of the patients.

	Patient 1	Patient 2	Patient 3
Gender	Female	Male	Male
Age (year)	73	48	63
Modified Oxford Scale score	5	5	5
Thickness of the left puborectalis muscle (mm)	9.4	9.0	8.3
Thickness of the right puborectalis muscle (mm)	6.9	8.8	5.4
Clinical stage	IIIA	IIIB	IIIC
T	2	3	4a
N	1	1	2
M	0	0	0
Tumor size (cm)	8.4	3.8	9.6
R0 resection	Yes	Yes	Yes
Distance of the anal verge (cm)	<1	3	2.3
Pathological stage	IIIB	IIA	IIIC
T	2	3	4b
N	2	0	2
M	0	0	0
Histological differentiation	Moderately	Moderately	Poorly
Perineural invasion	No	Yes	Yes
Vascular tumor thrombus	Yes	Yes	No
Tumor budding degree	2	1	2

### 3.1 Patient 1

A 73-year-old female was admitted owing to a “protruding anal mass for 2 h.” Colonoscopic findings suggested a circumferential mass extending from the anal verge to 8 cm inside the anus, involving the anus. DRE confirmed that the lower margin of the tumor was approximately 2 cm below the dentate line. Histopathological examination of the colonoscopy biopsy specimen revealed a moderately differentiated adenocarcinoma of the rectum. On March 21, 2023, the patient underwent PPFMR with ISAR (including complete IAS removal and a small portion of the EAS). The postoperative appearance of the anal region is shown in Figure 2. Chemotherapy with the mFOLFOX6 regimen was administered for two cycles between April 2023 and May 2023. Subsequently, owing to the patient's refusal to receive chemotherapy, the medication was discontinued. At 24 months postoperatively, follow-up imaging reveals no significant signs of recurrence or metastasis. The Wexner score and Low Anterior Resection Syndrome score of patient 1 were 7 and 18 (Table 2*)*, and she reported effective control of bowel movements.

**Table 2:** Postoperative wexner score and LARS score of three patients.

	1 month	3 months	6 months	12 months	18 months	24 months
**Wexner incontinence scores**	16/16/16 (P1/P2/P3)	12/16/16 (P1/P2/P3)	9/16/16 (P1/P2/P3)	9/16/16 (P1/P2/P3)	8/16/16 (P1/P2/P3)	7/12/16 (P1/P2/P3)
**LARS scores**	41/41/41 (P1/P2/P3)	37/41/41 (P1/P2/P3)	27/41/41 (P1/P2/P3)	27/41/41 (P1/P2/P3)	18/39/39 (P1/P2/P3)	18/37/37 (P1/P2/P3)

LARS, low anterior resection syndrome; P1, patient 1; P2, patient 2; P3, patient 3.

### 3.2 Patient 2

A 48-year-old male patient was admitted to the hospital in December 2020 because of “hematochezia.” Colonoscopy revealed a nodular mass obstructing the lumen at a distance–3–8 cm from the anal verge. DRE confirmed that the lower edge of the tumor was located 1 cm below the dentate line. Histopathological examination of the colonoscopy biopsy specimen revealed a moderately differentiated adenocarcinoma of the rectum. On December 15, 2020, the patient underwent a combined procedure of PPFMR with ISAR (including complete removal of the IAS and EAS) and *in situ* reconstruction of the anus. From December 25, 2020 to June 15, 2021, the patient underwent a total of six cycles of XELOX chemotherapy. Subsequent imaging examinations performed until 2025 did not reveal any signs of tumor recurrence or metastasis. At 24 months postoperatively, the Wexner score and Low Anterior Resection Syndrome score of patient 2 were 12 and 37 (Table 2*)*. His bowel function had significantly improved, with only one to two incidents of fecal incontinence per week, which did not substantially impact his daily life.

### 3.3 Patient 3

A 63-year-old male patient was admitted on September 14, 2021 because of “altered bowel habits for over 4 months.” Colonoscopy examination indicated the presence of a mass measuring approximately 1.8 × 2.0 cm at the anal entrance, and mucosal erosion was observed at the anal opening, extending to the rectal area approximately 10 cm from the anal verge. Preoperative pathological examination indicated poorly differentiated adenocarcinoma of the anus. Preoperative pelvic MRI revealed a thickness of 8.3 mm in the left PM and 5.4 mm in the right PM. On September 28, 2021, the patient underwent PPFMR with ISAR (including complete IAS and EAS removal and partial PM removal). The patient underwent seven cycles of mFOLFOX6 chemotherapy from October 2021 to May 2022. However, the tumor marker levels remained elevated. Consequently, the chemotherapy regimen was switched to FOLFIRI. Between May and June 2022, the patient received two cycles of the FOLFIRI regimen. Owing to intolerable toxicity associated with the chemotherapy drugs, treatment was discontinued. Radiotherapy was initiated on June 23, 2022. The specific protocol involved a total dose of 45 Gy delivered in 25 fractions over 5 weeks. Concurrently, the patient received capecitabine (1.5 g, po, bid) from Monday to Friday during radiotherapy. A contrast-enhanced pelvic CT scan performed on January 31, 2023 indicated peritoneal and abdominal wall metastases. Subsequently, combined TAS-102 and fruquintinib was administered. At 24 months postoperatively, the Wexner score and Low Anterior Resection Syndrome score of patient 3 were 16 and 37 (Table 2*)*. He could perceive the urge to defecate and suppress it for up to 20 s.

## 4 Discussion

Petros et al. (15) contended that the integral pelvic floor is a closely interrelated entity and that its complete functionality is achieved through the mutual coordination of the PFMs, connective tissues, and pelvic organ structures. This represents a unified collaboration between the supportive and sphincteric muscle systems. The PM, being a circular skeletal muscle, leads us to hypothesize that under the circumstance of partial excision that does not compromise its entire circumferential structure and innervating nerves, its functionality should be largely preserved. This hypothesis was validated in patients 1 and 2 who were assessed for well-functioning PFMs. In patients 1 and 2, in whom the complete PM was preserved, postoperative defecation function was significantly better than that in patient 3, who had a partial excision of the PM. Patient 3 experienced frequent fecal incontinence, Patient 1 had almost no fecal incontinence, whereas patient 2 experienced fecal incontinence only 1–2 times per week. Besides, follow-up evaluations suggested that the broader the resection of the PFMs, the more suboptimal the defecatory function. In patient 1, part of the EAS was retained, whereas in patient 2, the entire anal sphincter was excised. During postoperative follow-up, patient 1's defecation function was significantly superior to that of patient 2. The same applies to ISR procedures; the more extensive the removal of the anal sphincter, the more impaired the defecatory function after surgery (16). Therefore, while ensuring R0 resection, the extent of PFM excision should be precisely determined to achieve optimal postoperative defecation function. This may require a decision based on the tumor's preoperative T stage and degree of intraoperative extramural invasion. As of June 2025, three patients showed no signs of metastasis or recurrence. The long-term efficacy awaits further follow-up.

We adhered to strict criteria when selecting patients for combined PPFMR and ISAR procedures. Based on the PM thickness reported by Singh et al. (17), the average thickness of the left and right PM was approximately 6.5 ± 2.04 mm and 4.9 ± 2.3 mm, respectively. Only those patients meeting the following criteria were deemed eligible for undergoing combined procedures of PPFMR with ISAR: a left PM thickness of ≥6·5 mm, a right PM thickness of ≥4·9 mm, and a modified Oxford score of ≥5 levels. Moreover, owing to the uncertainty surrounding postoperative outcomes, we exclusively performed PPFMR with ISAR in patients whose desire to retain their anus far surpassed their consideration for life itself after fully informing them of the pertinent postoperative risks. Simultaneously, a contingency plan should be prepared; in the event that postoperative anal function does not meet the required criteria, subsequent APR surgery should be performed. This encompasses the closure of the anus along with the colostomy-creation procedure, which constitutes a relatively straightforward surgical process.

In principle, patients with advanced-stage rectal cancer must receive neoadjuvant therapy preoperatively to shrink the tumor, thereby increasing the likelihood of preserving the anus and reducing local recurrence rates (5). However, neoadjuvant chemoradiotherapy can induce fibrosis in rectal and anal tissues, thereby significantly increasing the incidence of fistulas at the anastomotic site (18, 19). Compared to postoperative radiotherapy, neoadjuvant chemoradiotherapy may lead to more severe rectal and anal functional impairments (20), without a corresponding improvement in the 10-year overall survival (21). Considering all these, the patients undergoing PPFMR with ISAR did not undergo neoadjuvant chemoradiotherapy. Postoperative radiotherapy was administered based on individual circumstances, as exemplified in patient 3.

Presently, the assessment criteria for postoperative anal function include Wexner incontinence and LARS scores. Both scoring systems have limitations as they do not fully account for the practical feelings and needs of postoperative patients in their daily lives and their acceptance of defecation function. Therefore, we propose the concept of “acceptable postoperative defecation function (APDF)” defined as follows. First, the patient perceives the urge to defecate, can exert a certain degree of control over bowel movements, and has the time to address defecation matters, such as waiting until reaching a restroom. This essentially fulfills the requirements of daily life. Second, defecation function is reduced to some extent postoperatively; however, compared to having a colostomy bag, patients are satisfied with their postoperative defecation function.

According to data from current studies, patients with rectal cancer who received type IV ISR surgery (including complete resection of the IAS and partial resection of the EAS) achieved an average Wexner score of 9.0–11.1 points at a 5-year postoperative follow-up (22, 23). Patient 1 achieved a notable Wexner score of 7 points at 24 months postoperatively. She is an elderly retired woman who rarely ventures outside and generally remains within the confines of her residential community. She reported effective control of bowel movements with virtually no fecal incontinence and no need for pads. The surgical impact on the patient's daily life was minimal, and the results were remarkable. Although patients 2 and 3 experienced poor defecation function in the short-term after surgery, defecation function improved to some extent after 24 months of follow-up. Patient 2 achieved a Wexner score of 12 points, which was close to the average level observed after IV-type ISR surgery. He reported being able to suppress the urge to defecate for 3–5 min, allowing sufficient time to reach a restroom. However, his stools were occasionally unformed, and he experienced fecal incontinence one to two times per week. During business travel, the patient adopted a low-fiber diet to minimize stool output and used protective pads. Patient 3 had a Wexner score of 16. He is an elderly retired man who spends most of his time within his residential community. His daily needs were primarily addressed by his wife, although he was capable of living independently when she was not present. He reported that he was able to perceive the urge to defecate and could suppress it for up to 20 s. His bowel movements did not follow a fixed schedule, and episodes of fecal incontinence were frequent, necessitating the use of protective pads throughout the day. However, he occasionally reached the restroom during defecation. All three patients expressed overall satisfaction with their postoperative defecation function. This outcome confirms our hypothesis that patients with ULRC/ACC with good PFM function can maintain a satisfactory level of what we define as APDF after undergoing combined PPFMR with ISAR.

Currently, no surgical procedures ensure the perfect preservation of anal function. Among patients who undergo ISR, especially type IV ISR, a significant proportion who experience postoperative anal dysfunction or even anal incontinence remains (23). Part of the reasons may also be associated with the lack of rigorous preoperative assessment of patients' PFM functionality. In carefully selected cases, our surgical approach enabled patients to achieve APDF. Our surgical approach, combined PPFMR with ISAR, enables patients to attain APDF, with short-term oncological outcomes no worse than those of traditional APR surgery (24).

Patients with good pelvic floor function who strongly refuse permanent stoma can undergo a combination of partial pelvic floor muscle resection and *in situ* anal reconstruction. This enables a greater number of patients with ULRC or ACC to undergo curative oncologic surgery while preserving the APDF, thereby enhancing their overall quality of life. We will continue to expand the sample size and collaborate with other affiliated hospitals to conduct a multicenter prospective study. Patients will be further classified based on their varying lifestyles and tumor conditions to establish a comprehensive evaluation system to ensure quality of life following surgery.

## Data Availability

The raw data supporting the conclusions of this article will be made available by the authors, without undue reservation.
